# miR-548j-5p regulates angiogenesis in peripheral artery disease

**DOI:** 10.1038/s41598-022-04770-6

**Published:** 2022-01-17

**Authors:** Chiu-Yang Lee, Shing-Jong Lin, Tao-Cheng Wu

**Affiliations:** 1 Division of Cardiovascular Surgery, Department of Surgery, Taipei Veterans General Hospital, Taipei, Taiwan; 2 Institute of Clinical Medicine, School of Medicine, National Yang Ming Chiao Tung University, Taipei, Taiwan; 3 Division of Cardiology, Department of Medicine, Taipei Veterans General Hospital, No. 201, Section 2, Shih-Pai Road, Taipei, 112 Taiwan; 4 Cardiovascular Research Center, National Yang Ming Chiao Tung University, Taipei, Taiwan; 5 Taipei Heart Institute, Taipei Medical University, Taipei, Taiwan; 6 Department of Medical Research, Taipei Veterans General Hospital, Taipei, Taiwan

**Keywords:** Stem cells, Cardiology

## Abstract

Peripheral artery disease (PAD) is a vascular disease involving diffuse atherosclerosis, and is associated with increased cardiovascular mortality and morbidity. Critical limb ischemia (CLI) is the most severe complication of PAD. In addition to medical and interventional treatment, therapeutic angiogenesis is a novel therapy for PAD. Circulating microRNAs (miRNAs) are considered key regulators of gene expression, but their role in ischemic-induced angiogenesis is poorly-characterized. There is currently a limited understanding of the specific miRNAs associated with PAD. To determine the regulation of miRNAs, we obtained miRNA profiles using RNA isolated from patients with PAD and a control group. The effects of specific miRNAs on angiogenesis were evaluated by assessing the in vitro angiogenic function of endothelial progenitor cells (EPCs), performing an in vivo angiogenesis assay, and employing a mouse hindlimb ischemic model. Our results demonstrated that circulating miR-548j-5p was significantly reduced in patients with PAD as compared with the controls. miR-548j-5p promoted EPC angiogenesis by enhancing migration and tube formation. The endothelial nitric oxide synthase (NOS) and stromal cell-derived factor (SDF)-1 signaling pathways appeared to be potential targets of miR-548j-5p. Furthermore, the results of a directed in vivo angiogenesis assay of EPCs and a hindlimb ischemia mouse model demonstrated that miR-548j-5p enhanced the capillary density and blood flow recovery in hindlimb ischemia. In conclusion, our data indicated that up-regulation of miR-548j-5p promotes angiogenesis in ischemic tissue and may represent a novel therapeutic approach for PAD.

## Introduction

Peripheral artery disease (PAD) is an atherosclerotic disease that increases the risk of cardiovascular mortality and morbidity^1^. The prevalence of PAD is greater than 20% in individuals over 60 years of age, and incidence rates have been predicted to increase with aging populations^2^. Critical limb ischemia (CLI) is the most severe clinical presentation of PAD, and causes intermittent claudication, ulceration and gangrene of the foot; it is associated with amputation and cardiovascular death^3^. The pathophysiology of PAD is a complex process involving endothelial dysfunction, oxidative stress, platelet activation and inflammation^4^. Despite pharmacological control of risk factors, the clinical outcomes of patients with PAD are poor. Thus, the development of new therapeutic targets for patients with PAD is an important issue.

Recently, microRNAs (miRNAs) have emerged as novel regulators of vascular biology. miRNAs are small, non-coding RNAs that regulate gene expression at the post-transcriptional level to repress expression^5^. miRNAs play key roles in inflammation, angiogenesis, endothelial function and restenosis associated with PAD^6^. Previous studies have shown that specific miRNA expression profiles in patients with PAD may serve as prognostic predictors^7^. In addition, some miRNAs, such as miR-93^8^ and miR-let-7 g^9^, have been shown to mediate angiogenesis through various molecular pathways. However, the associations between miRNAs and PAD have not yet been fully characterized.

There is some evidence to indicate that endothelial progenitor cells (EPCs) are involved in angiogenesis in patients with postmyocardial infarction^10^ and limb ischemia^11^. It has also been reported that miRNAs can regulate the proliferation^12^, migration^13^ and tube formation^14^ of EPCs. These findings suggest that abnormal expressions of miRNAs are involved in different cardiovascular diseases, such as atherosclerosis, coronary and peripheral artery diseases. Recently, increasing evidence has shown that miRNAs can be used as diagnostic biomarkers for cardiovascular diseases^15^. However, in order to identify the mechanisms underlying regulation of the progress of PAD by miRNAs, additional studies are required to validate miRNAs with the potential for use in the treatment of this class of diseases.

The aim of the present study was to explore specific miRNAs relevant to PAD and investigate their mechanisms in the disease, and then further investigate the angiogenic effects of these miRNAs on EPC migration, tube formation and angiogenesis in PAD. The results of our study could provide useful information regarding the utilization of miRNAs as a novel therapeutic strategy for patients with PAD.

## Results

### Detection of circulating miRNAs

The baseline demographic and clinical characteristics of the PAD group and control group are shown in Table 1. In total, 40 subjects from Taipei Veterans General Hospital, Taipei, Taiwan, were studied, 25 patients with documented PAD and 15 individuals without evidence of PAD (control group). The age of the PAD group and control group was 74 ± 7.1 years and 44 ± 14.9 years, respectively. There were 17 male patients in the PAD group and 13 male subjects in the control group. The median body mass index (BMI) of the PAD group was 23.19 ± 2.82, and that of the control group was 25.5 ± 0.64. There were 19 patients with hypertension and 15 with diabetes mellitus in the PAD group. Coronary artery disease and chronic kidney disease were present in 5 and 10 patients in the PAD group, respectively.

**Table 1:** Baseline characteristics of the study population.

	PAD group (*n* = 25)	Control group (*n* = 15)
**Demographics**		
Age (years)	74 ± 7.1	44 ± 14.9
Male sex	17 (8)	13 (2)
BMI	23.19 ± 2.82	25.5 ± 0.64
**Risk factors**		
Hypercholesterolemia	3 (22)	0
Hypertension	19 (6)	1 (14)
Diabetes mellitus	15 (10)	1 (14)
Coronary artery disease	5 (20)	0
Chronic kidney disease	10 (15)	0
Smoking	8 (17)	1 (14)
**Medications**		
Aspirin	9 (16)	0
Clopidogrel	9 (16)	0
Statins	9 (16)	0
ACE inhibitors	3 (22)	0
ARB	10 (15)	1 (14)
β-blockers	10 (15)	0
Calcium channel blockers	12 (13)	0
**Clinical presentation**		
Fontaine classification		
Stage III	10	0
Stage IV	15	0

**Figure 1:**
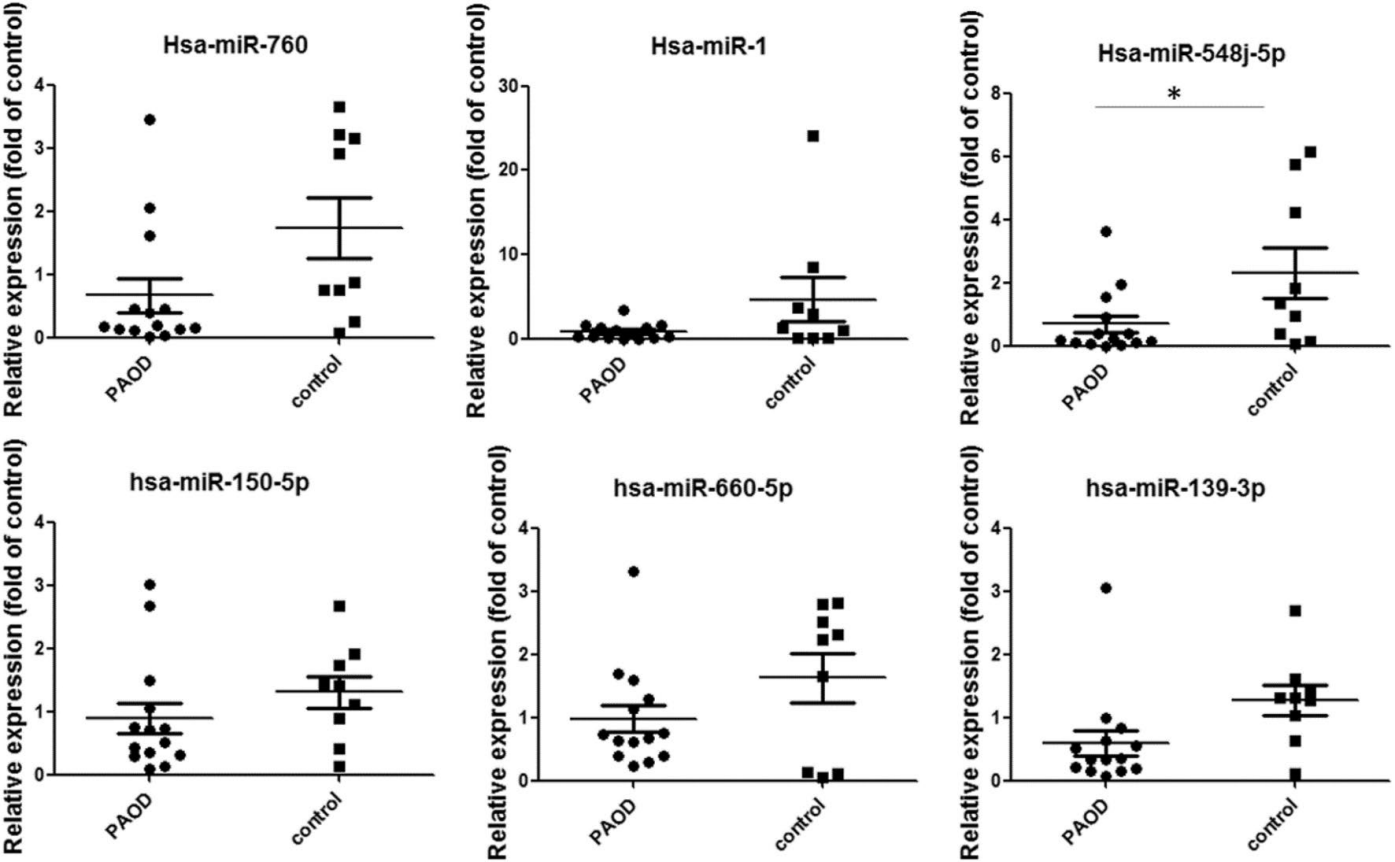
Expressions of microRNAs in patients with PAD as compared with the control group. Candidate miRNAs in the PAD group and the control group were analyzed. miR-548j-5p was significantly decreased in patients in the PAD group as compared with the controls (*P < 0.05 vs. control group).

The concentrations of several circulating miRNAs were measured, and the PAD group had a significantly decreased expression of miR-548j-5p (AAAAGUAAUUGCGGUCUUUGGU) as compared with the control group (Fig. 1).

### miR-548j-5p enhances tube formation and migration in EPCs

**Figure 2:**
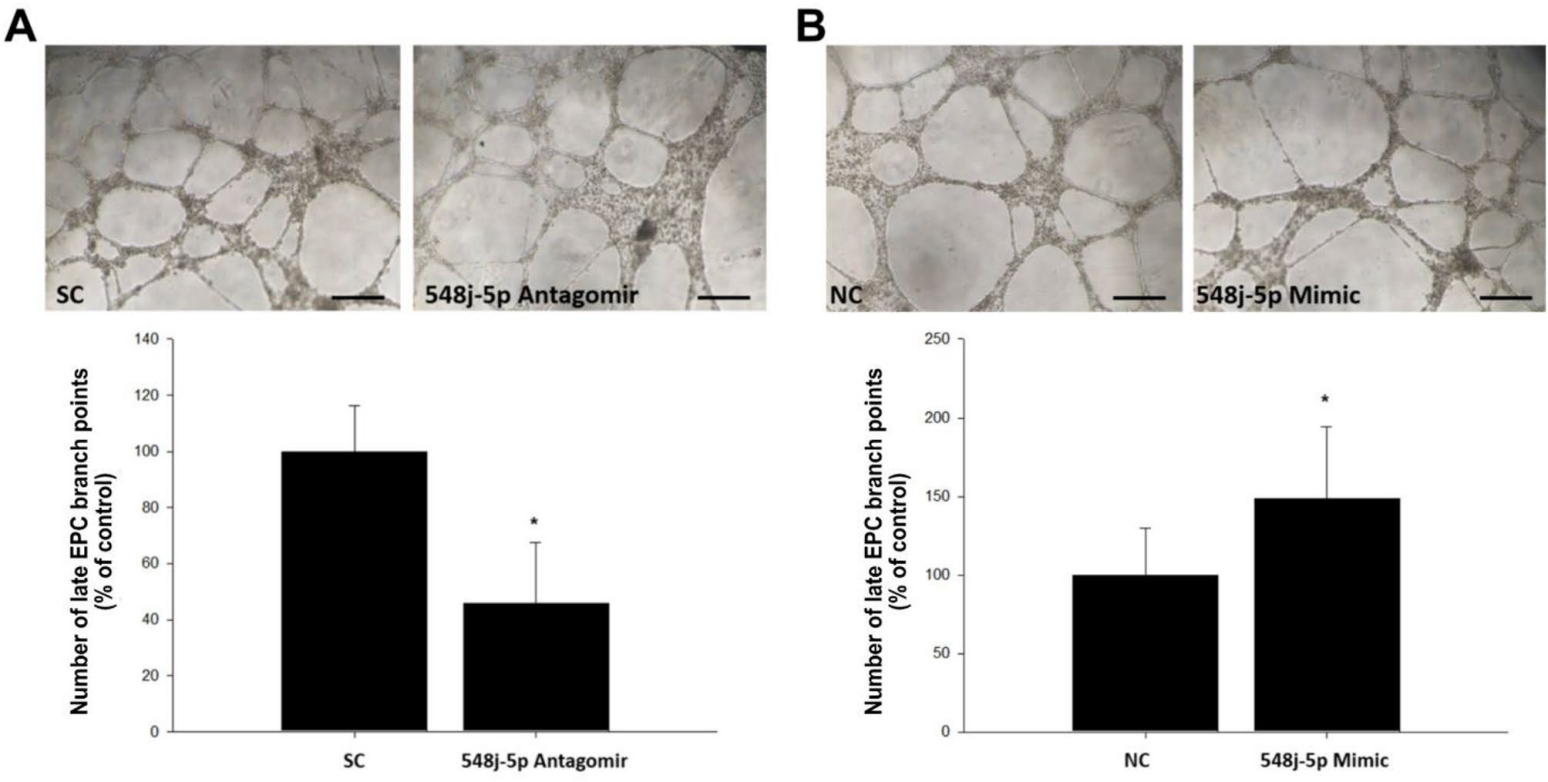
Effect of miR-548j-5p on human EPC tube formation. An in vitro angiogenesis assay of EPCs was performed using ECMatrix gel. (**A**) Human EPCs transfected with miR-548j-5p antagomir exhibited impaired tube formation as compared with the miR-scramble control (SC) in vitro; therefore, miR-548j-5p under-expression in EPCs could inhibit EPC tube formation. (**B**) Human EPCs transfected with miR-548j-5p mimic exhibited enhanced tube formation as compared with the miR-mimic negative control (NC) in vitro, meaning that miR-548j-5p over-expression in EPCs could promote EPC tube formation. Data are presented as the mean ± SEM (*P < 0.05 vs. control, *n* = 6 for each experiment).

Tube formation and mobilization in EPCs in the ischemic area are important for neovascularization. The effect of miR-548j-5p on tube formation in EPCs was investigated, as shown in Fig. 2. Transfection of miR-548j-5p mimics into EPCs significantly increased their tube formation as compared with the control group.

**Figure 3:**
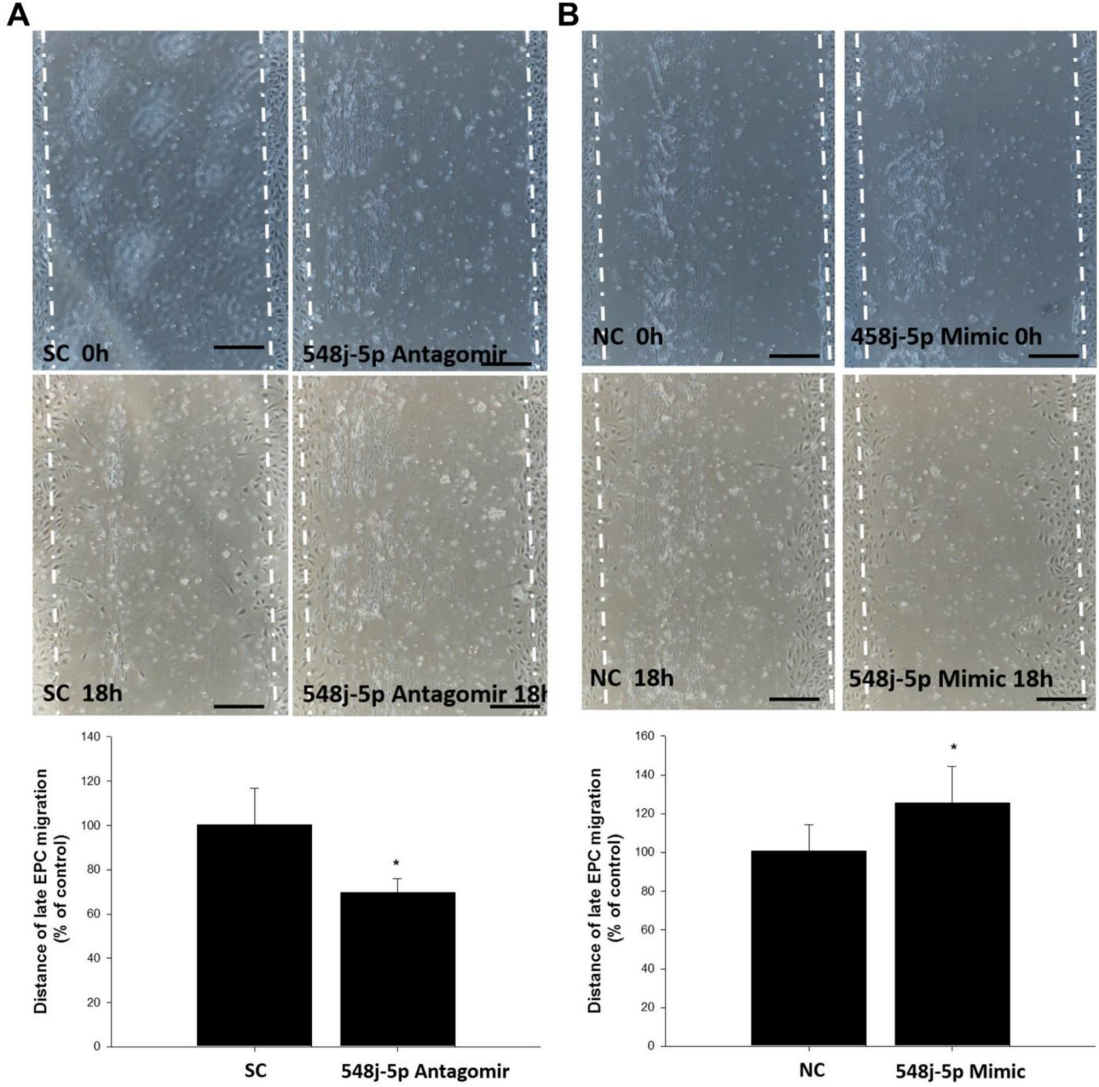
Effect of miR-548j-5p on human EPC migration. The migratory function of EPCs was evaluated using a scratch injury model. (**A**) Human EPCs transfected with miR-548j-5p antagomir exhibited suppressed EPC mobilization as compared with the miR-scramble control (SC) in vitro; this means that miR-548j-5p under-expression in EPCs could inhibit EPC migration. (**B**) Human EPCs transfected with miR-548j-5p mimic exhibited enhanced EPC mobilization as compared with the miR-mimic negative control (NC) in vitro, meaning that miR-548j-5p over-expression in EPCs could promote EPC migration. Data are presented as the mean ± SEM (*P < 0.05 vs. control, *n* = 6 for each experiment).

Compared with the control group, transfection of miR-548j-5p mimics into EPCs significantly increased their migration, as shown in Fig. 3; however, transfection of miR-548j-5p antagomir into EPCs decreased their migration. Thus, miR-548j-5p could modulate the angiogenic activities of EPCs.

### miR-548j-5p promotes the angiogenic potential of EPCs according to a directed in vivo* angiogenesis assay (DIVVA)*

**Figure 4:**
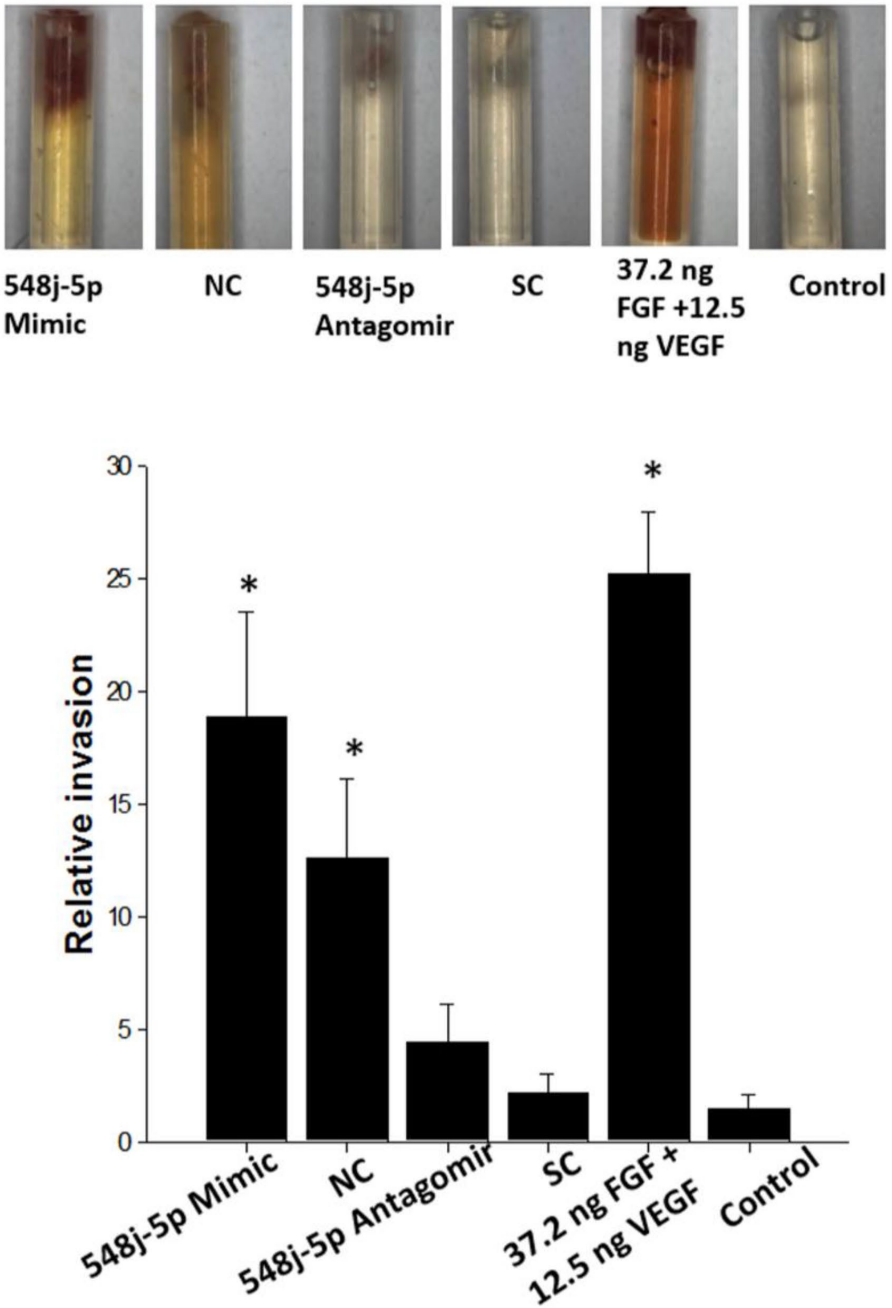
Effect of miR-548j-5p on blood vessel formation in vivo, assessed using a directed in vivo angiogenesis assay. Representative images of angioreactors removed from nude mice 9 days after implantation showed that miR-548j-5p enhanced blood vessel formation. While FGF-2 and VEGF have both been demonstrated to promote angiogenesis in DIVAA, the FGF/VEGF mixture provides a synergistic effect, allowing a drastic increase in response using a lower growth factor concentration. 548j-5p Mimic: transfected with miR-548j-5p mimic; NC: transfected with miR-mimic negative control; 548j-5p Antagomir: transfected with miR-548j-5p antagomir; SC: transfected with miR-scramble control. Data are presented as the mean ± SEM (*P < 0.05 vs. control, *n* = 6 for each experiment).

The angiogenic effects of miR-548j-5p were further analyzed using a DIVVA, as shown in Fig. 4. Transfection of miR-548j-5p mimics into EPCs significantly increased the invasion of vessels in the angioreactors as compared with the negative control. However, transfection of miR-548j-5p antagomir into EPCs decreased the invasion of vessels in the angioreactors as compared with the scramble control. While FGF-2 and VEGF have both been demonstrated to promote angiogenesis in DIVAAs, the FGF/VEGF mixture provides a synergistic effect, allowing a drastic increase in response at a lower growth factor concentration. These findings indicated that miR-548j-5p could promote the angiogenic capacity of EPCs.

### miR-548j-5p regulates EPC function via* targeting of eNOS and SDF-1*

**Figure 5:**
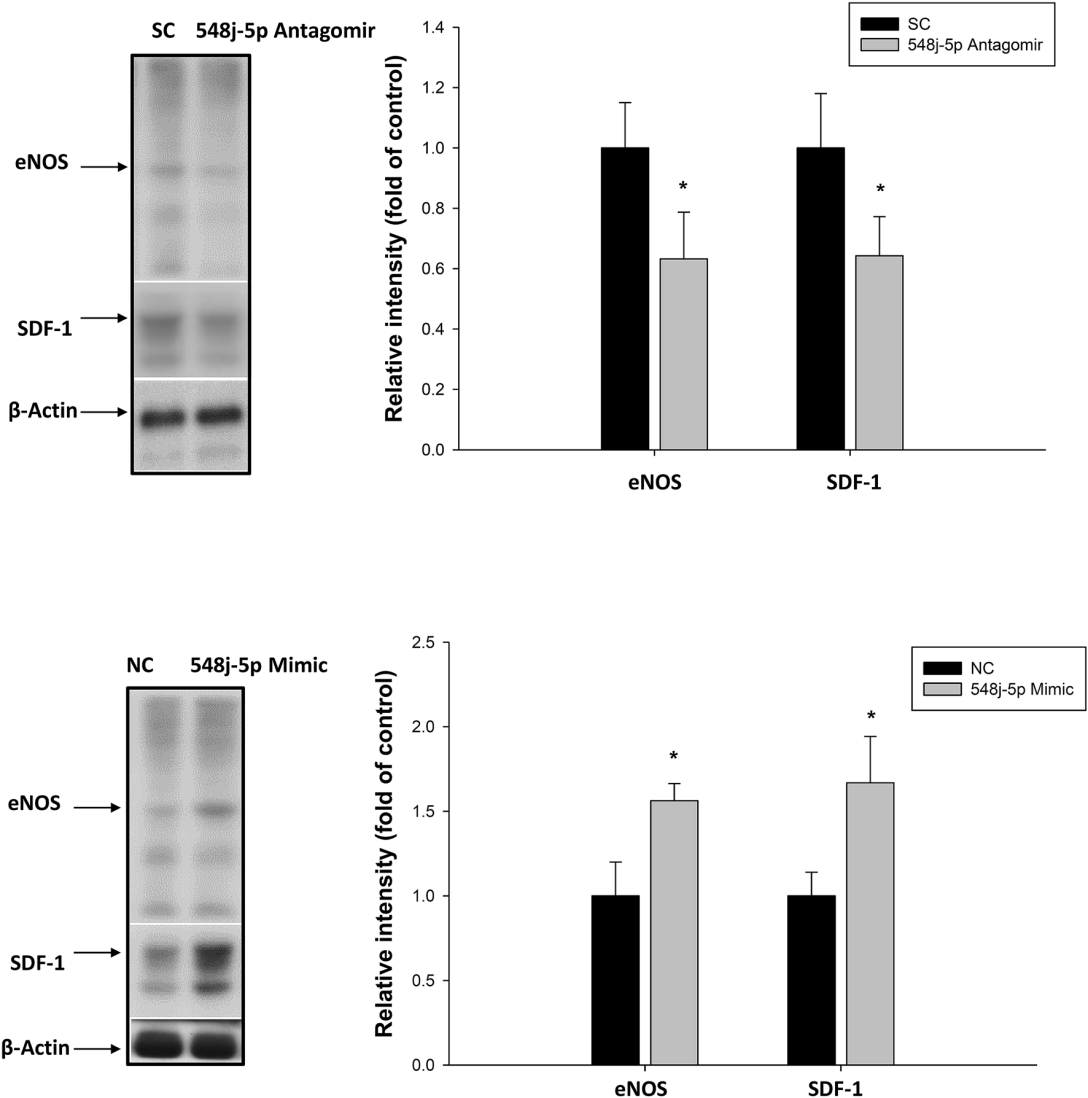
Effects of miR-548j-5p on eNOS and SDF-1 production in EPCs. Impaired expressions of eNOS and SDF-1 were observed in EPCs transfected with miR-548j-5p antagomir as compared with the miR-scramble control (SC). Enhanced expressions of eNOS and SDF-1 were observed in EPCs transfected with miR-548j-5p mimic as compared with the miR-mimic negative control (NC). Data are presented as the mean ± SEM (*P < 0.05 vs. control, *n* = 6 for each experiment). (Full-length blots/gels are presented in Supplementary Fig. 1.)

miR-548j-5p antagomir transfection of EPCs downregulated the eNOS and SDF-1 expressions, as shown in Fig. 5. However, EPCs with miR-548j-5p mimic transfection exhibited a significantly upregulated eNOS expression. In addition, miR-548j-5p mimic transfection of EPCs upregulated the SDF-1 expression. These results suggested that miR-548j-5p could regulate EPC function via the eNOS and SDF-1 signaling pathways.

### miR-548j-5p promotes blood flow recovery in hindlimb ischemia in a mouse model

**Figure 6:**
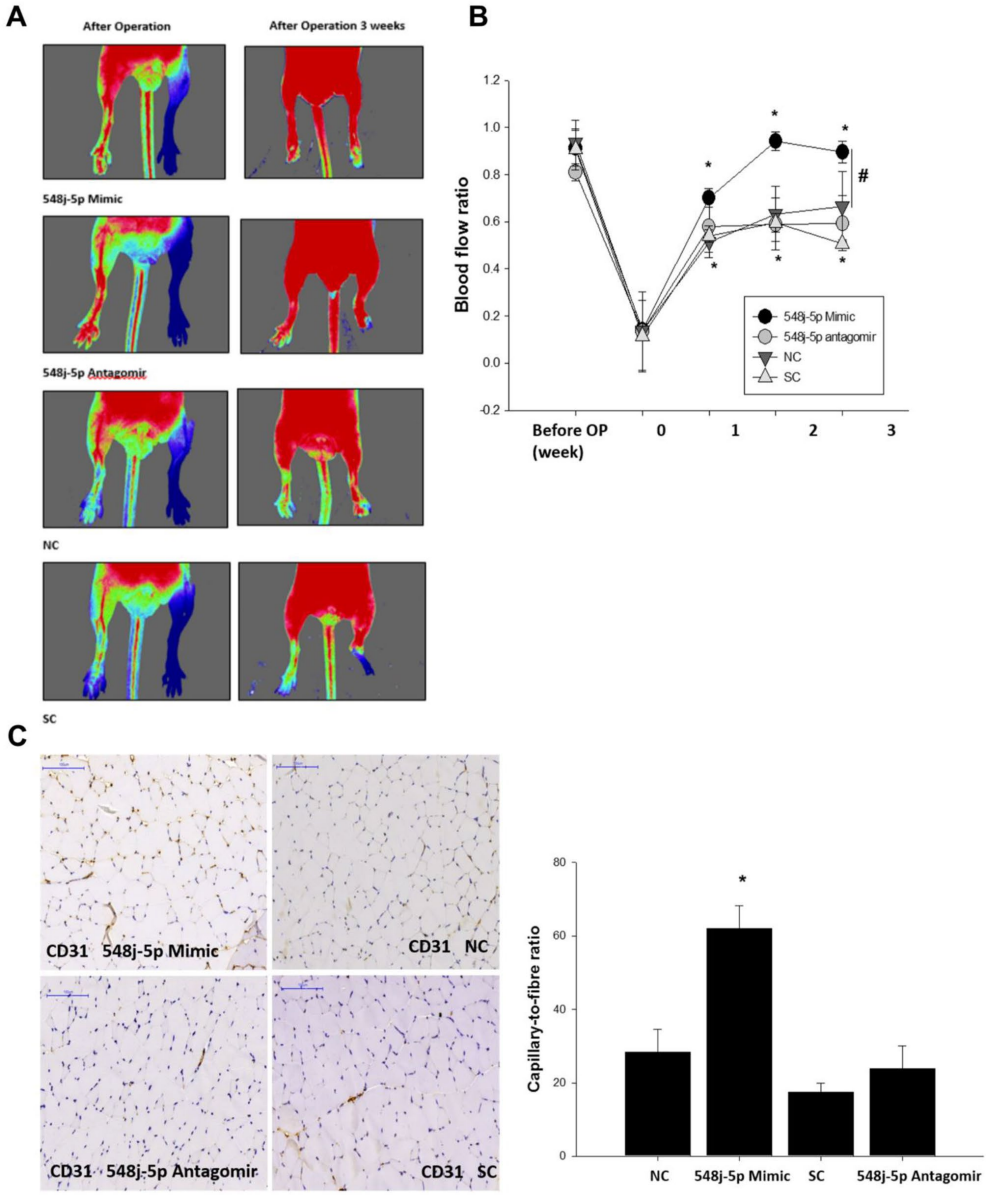
Effect of miR-548j-5p on hindlimb perfusion. (**A**) Serial Doppler analysis of hindlimb perfusion before and 3 weeks after hindlimb ischemia surgery in nude mice that received transplants of EPCs transfected with miR-548j-5p mimic, miR-548j-5p antagomir, miR-mimic negative control (NC) or miR-scramble control (SC). The color scale illustrates blood flow variation from minimal (dark blue) to maximal (red) values. Arrows indicate the ischemic limb after hindlimb ischemia surgery. (**B**) Quantification of perfusion recovery using the laser Doppler perfusion imaging ratio (ischemia/normal hindlimb) in the different groups. (**C**) Mice were sacrificed 3 weeks after surgery, and capillaries in the ischemic muscles were visualized using anti-CD31 immunostaining. Data are presented as the mean ± SEM (*P < 0.05 vs. NC; ^#^*P* < 0.05 vs. miR-548j-5p antagomir, *n* = 6 for each experiment).

To evaluate the angiogenic effect of miR-548j-5p, a mouse hindlimb ischemia model was developed. Compared with the control group, the mice with miR-548j-5p mimic transfection of EPCs exhibited significantly enhanced flow recovery; however, the mice with miR-548j-5p antagomir transfection showed delayed blood flow recovery after surgery (Fig. 6A and B). Anti-CD31 immunostaining showed a decreased capillary density in mice with miR-548j-5p antagomir transfection of EPCs as compared with the control mice, but the miR-548j-5p mimic mice exhibited a significantly increased capillary density in muscles (Fig. 6C). Therefore, miR-548j-5p could enhance the angiogenic activities of EPCs.

## Discussion

To the best of our knowledge, this was the first study to document down-regulation of miR-548j-5p in PAD. Using an in vivo angiogenesis assay, this study demonstrated that transfection of EPCs with miR-548j-5p could enhance the activities of eNOS and SDF-1 and improve EPC functions such as migration and tube formation, whereas miR-548j-5p antagomir transfection impaired the angiogenic activities of EPCs. Using a mouse model of hindlimb ischemia, we further showed that transfection of EPCs with miR-548j-5p improved blood flow recovery. These results suggested that miR-548j-5p plays an important role in the processes associated with angiogenesis.

Recently, circulating miRNAs have been shown to serve as novel biomarkers for cardiovascular disease^15,16^. Previous reports have indicated the importance of miRNAs in the regulation of angiogenic functions of progenitor and stem cells in the cardiovascular system^17^. Alterations of the levels of several miRNAs in the serum are thought to be linked to the development of several cardiovascular diseases, suggesting that these miRNAs represent prospective therapeutic targets^15^. There are two major therapeutic approaches to the use of miRNAs in clinical application: up-regulation of miRNAs for therapeutic purposes, and mimicking the functions of endogenous miRNAs^18^.

In patients with PAD, individual miRNAs have been found to be differentially-expressed in circulation. Using next-generation sequencing to investigate miRNA and gene expression profiles in patients with lower-extremity arterial disease, Bogucka-Kocka et al*.*^19^ identified dysregulation of 26 miRNAs that may be used as novel biomarkers for the disease.

Higher levels of circulating miR-15a and miR-16 have been observed in CLI patients with and without type 2 diabetes mellitus as compared with healthy controls, and the two miRNAs predicted a poorer prognosis for those patients with diabetes mellitus^20^. Using phenotypically different inbred mouse strains of murine hindlimb ischemia, Hazarika et al*.*^21^ found that miR-93 was differentially-expressed in the hindlimb ischemia mice, and in vivo study further demonstrated that overexpression of miR-93 enhanced cell proliferation and endothelial cell tube formation, which suggested that miR-93 modulates ischemic-induced angiogenesis independent of HIF-1α-regulated angiogenic genes.

miR-146a has been found to be a regulator of vascular remodeling. Heuslein et al*.*^22^ reported that suppression of miR-146a enhanced arteriogenesis in the muscular collateral circulation via upregulation of pro-inflammatory endothelial activation. Metalloproteinase domain-containing protein 12 (ADAM12) has been identified as a key gene related to modification of PAD severity in mice^23^, and forced miR-29a expression inhibited the ADAM12 expression in ischemic endothelial cells; in addition, knock-down of miR-29a improved the impaired post-ischemic angiogenesis in a diabetic mouse model^24^. In the present study, we demonstrated that miR-548j-5p facilitates cell migration and tube formation in EPCs, suggesting that miR-548j-5p contributes to angiogenesis in PAD by regulating the angiogenic activities of EPCs.

miRNAs may function as mediators in the pathophysiology of CLI. It is known that different miRNAs may target the same mRNA or different targets from the same signaling pathway; thus, cooperative function of miRNAs has been proposed as a new therapeutic tool, as synergistic regulation of different features of impaired cellular signaling may be of benefit in the treatment of patients with PAD^25^. However, in order to realize this therapeutic approach, it is necessary to understand the mechanisms and the pathways involved for individual potential miRNAs. For example, miR-24-3p was reported to regulate angiogenesis by inhibition of the expression of STAT3^26^, a regulator of angiogenesis beyond inflammation^27^, and the expression of miR-24-3p in human blood was reported to be negatively correlated with the ankle brachial index (ABI)^26^.

The mechanisms of long non-coding RNA (lncRNA) and miRNA interaction in angiogenesis represent another interesting topic. Numerous studies have shown that lncRNA may serve as an endogenous sponge to regulate the expressions and functions of miRNAs^28^. For example, lncRNA/miRNA biomarkers of specific cancers have been identified based on this method^29^. As enhancement of angiogenesis to promote limb blood flow is key for the treatment of PAD, the knowledge gained regarding angiogenesis in cancer research may also be of benefit in terms of identifying candidate lncRNA/miRNA biomarkers for PAD.

Nitric oxide (NO) plays a key role in angiogenesis in ischemic disease, and eNOS synthetization of NO could promote migration of EPCs^30,31^. SDF-1 is a chemokine that has a strong chemotactic effect on EPCs. After vascular injury, SDF-1 induces migration of EPCs to the injured area and promotes endothelialization^32,33^. Under hyperglycemic conditions, miR-133a was found to be up-regulated, and induced endothelial dysfunction through inhibition of eNOS^34^. Chen et al*.*^35^ reported that the diabetes-induced expression of miR-133a impaired angiogenesis in PAD via a reduction in NO synthesis. In addition, miR-133a antagonism improved post-ischemic angiogenesis. Besides, EPCs may promote angiogenesis by releasing growth factors, which act in a paracrine manner to support local angiogenesis and mobilize tissue-residing progenitor cells. Angiogenic growth factors levels were higher in EPCs as compared with HUVECs; thus, the selection of EPCs in our study had greater clinical significance. In the current study, we found that up-regulation of miR-548j-5p induced the expressions of eNOS and SDF-1 in EPCs. These results revealed that miR-548j-5p enhanced migration and tube formation in EPCs by targeting the eNOS signaling pathway.

miRNAs have diverse functions in different contexts through regulation of different targets. In our study, we demonstrated that miR-548j-5p enhanced blood flow recovery in response to tissue ischemia. miR-548j-5p also improved EPC functions, such as migration, tube formation and sprouting, and enhanced the activities of eNOS and SDF-1. Our findings suggested that miR-548j-5p has beneficial effects on EPCs under hypoxic conditions and might provide vascular protection in the clinical setting.

Conservation of miR-548j-5p between humans and mice represents another interesting issue. A previous study demonstrated that 60% of mouse miRNA loci are conserved between mice and humans^36^ (Pal AS, Kasinski AL. Animal Models to Study MicroRNA Function. Adv Cancer Res. 2017;135:53–118). In our study, we used human EPCs transfected with human miR-548j-5p, which were then implanted in a hindlimb ischemic nude mouse model to evaluate the effects of human miR-548j-5p on angiogenesis. In order to ascertain the relationship of human miR-548j-5p with mouse miR-548j-5p, we searched the miRDB, which is an online database for miRNA target prediction and functional annotations. We found that there were 1596 predicted targets of hsa-miR-548j-5p in the miRDB, but no mouse miRNA was found for "548j-5p". This means that there is no conservation between human miR-548j-5p and mouse miR-548j-5p, and therefore it is not clear whether hsa-miR-548j-5p is conserved between humans and mice. However, there may be new research findings, such as the use of transgenic mouse models, to clarify the relationship between human miR-548j-5p and mouse miR-548j-5p in the future.

There were some limitations of our study. First, the in vitro and in vivo experiments were not conducted with control miRNAs. Second, owing to the small number of cases, the two groups were mismatched in terms of age, gender and risk factors. In the present study, the age difference between the PAD group and the control group was significant. It has been reported that age has a great influence on the expressions of miRNAs. Noren Hooten et al*.*^37^ reported significant down-regulation of miR-24, -130a,-155 and -221/222 in healthy older subjects as compared with young individuals, while Dhahri et al*.*^38^ found that miR-130a is associated with the modulation of endothelial cell senescence and impaired ischemia-induced neovascularization. These results indicate that miRNAs might play an important role in angiogenesis within the context of aging. In addition, increased expressions of miR-10A* and miR-21 in elderly subjects as compared with young subjects lead to decreased migration and proliferation of EPCs via an Hmga2(high-mobility group A)-dependent mechanism. EPCs in older subjects transfected with anti-miRs exhibited increased tube formation and improved blood flow recovery^39^. Additionally, miR-15 and miR-34 are linked to increased apoptosis and a reduced level of eNOS activity, and are associated with the senescence phenotype in human endothelial cells^40^. Study of a greater number of cases in the future is needed to confirm whether or not miR-548j-5p expression is age-dependent; however, the results of our in vitro and mouse models did indicate that miR-548j-5p plays a critical role in PAD. Therefore, a study in which age, gender and risk factors are matched should be performed in the future.

Third, in the present study, the circulating level and muscle level of miR-548j-5p were not analyzed. A functional assay of miR-548j-5p showed over-expression of miR-548j-5p in EPCs, which increased blood flow recovery and capillary density in hypoxic tissue. Fourth, SDF-1 and eNOS play important roles in angiogenesis in PAD. Correlations between miR-548j-5p and these two angiogenic proteins were observed in our study. A reporter assay of SDF-1 and eNOS will be performed in future study.

The results of the present study demonstrated that miR-548j-5p contributes to the pathological processes associated with angiogenesis by promoting migration and tube formation in EPCs, which are associated with the expressions of eNOS and SDF-1. Up-regulation of miR-548j-5p improved neovascularization in hindlimb ischemic mice.

Thus, miR-548j-5p may have therapeutic potential for PAD. Further studies are needed to evaluate the therapeutic effects and to examine the clinical implications of these findings.

## Methods

This research was conducted according to the principles expressed in the Declaration of Helsinki. The study was approved by the Institutional Review Board for Research of Taipei Veterans General Hospital (approval number: 2013–08-020B#3). Written informed consent was provided by all participants prior to their inclusion in the study.

The animal study protocol was approved by the Institutional Animal Care and Use Committee of Taipei Veterans General Hospital, Taipei, Taiwan (approval number: IACUC_2013–076) and was carried out in compliance with the ARRIVE guidelines.

### Study population

A total of 40 subjects were included in the current study, including 25 patients with PAD who were diagnosed according to an ankle brachial index (ABI) < 0.8 at screening or a previous revascularization of the lower extremity between November 2013 and December 2015. Patients with acute coronary syndrome, acute ischemic limb or malignancy were excluded. A total of 15 healthy individuals without any evidence of PAD served as the control group. Blood sampling was performed, and samples were subjected to RNA isolation (FocusGenomics Biotech Co., Ltd, Taiwan). Following miRNA array analysis, we selected specific miRNAs for comparison between the patients with PAD and the control group. This study was approved by the Institutional Review Board for Research of Taipei Veterans General Hospital (approval no.: 2013–08-020B#3), and written informed consent was provided by all the participants prior to their inclusion in the study.

### miRNA library construction and next-generation sequencing

RNA was isolated according to the manufacturer’s protocol. Whole blood (5 ml) was collected in an EDTA tube from the patients and centrifuged at 1,000 g for 5 min to collect plasma. Total RNA was isolated from plasma using QIAzol Lysis Reagent and purified using miRNeasy Kits (Qiagen GmbH) according to the manufacturer’s protocol. RNA quality and quantity were measured using a NanoDrop Spectrophotometer (ND-1000; NanoDrop; Thermo Fisher Scientific, Inc., Waltham, MA, USA). Total RNA from samples was further constructed into libraries using an Illumina TruSeq Small RNA Library Prep kit (Illumina, San Diego, CA, USA). In brief, 1 μg of high-quality total RNA from each sample was ligated with adapters using T4 RNA ligase. The adapter-ligated RNA samples were then reverse-transcribed to cDNA, amplified by primers containing a specific sequence index, and size-validated using an Agilent 2100 bioanalyzer (Agilent, Santa Clara, CA, USA) loaded with a DNA 1000 kit (Agilent). The size-checked libraries were loaded onto Novex TBE gels (Thermo Fisher Scientific, Inc.) and then size-selected and gel-eluted to obtain proper fragments. The eluted libraries were qualified using an Agilent 2100 bioanalyzer loaded with a DNA 1000 kit, and quantified using Qubit (Thermo Fisher Scientific, Inc.) and real-time PCR (qRT-PCR). Each library was diluted in equal concentrations and the same volumes were taken for pooling. Pooled libraries were sequenced for 10 M reads/sample with a high-throughput, 50-bp single-end sequencing reagent on an Illumina MiSeq sequencing system.

### qRT-PCR analysis

Total RNA was isolated from plasma samples and used as the input to assay the expressions of miR-760, miR-1-5p, miR-150-5p, miR-548j-5p, miR-660-5p, and miR-139-5p using TaqMan MicroRNA Assay kits (Applied Biosystems, Foster City, CA, USA) according to the manufacturer’s instructions. PCR reactions were carried out on a Quantica real-time nucleic acid detection system (Techne Inc., Burlington, NJ, USA) using miR-16 as the internal control. The comparative threshold cycle (Ct) method was used to measure relative change in expression; 2^ΔΔCt^ represented the fold change in expression. A negative control without a template was run in parallel to assess the overall specificity of the reaction. The primer information is presented below.

**Table:** 

hsa-miR-760	CGGCUCUGGGUCUGUGGGGA
hsa-miR-1-5p	ACAUACUUCUUUAUAUGCCCAU
hsa-miR-150-5p	UCUCCCAACCCUUGUACCAGUG
hsa-miR-548j-5p	AAAAGUAAUUGCGGUCUUUGGU
hsa-miR-660-5p	UACCCAUUGCAUAUCGGAGUUG
hsa-miR-139-5p	UCUACAGUGCACGUGUCUCCAGU
hsa-U6 snRNA	CGCAAGGATGACACGCAAATTC

### EPC isolation and cultivation

EPCs were cultured and identified according to the protocol described in a previous study (Wu TC, Chen JS, Wang CH, Huang PH, Lin FY, Lin LY, Lin SJ, Chen JW. Activation of heme oxygenase-1 by Ginkgo biloba extract differentially modulates endothelial and smooth muscle-like progenitor cells for vascular repair**.** *Sci Rep*. 2019; 9(1): 17,316).

Total mononuclear cells (MNCs) were isolated from 10 mL of peripheral blood obtained from the control volunteers and PAD patients (approval No.: 2013–08-020B#3) by density gradient centrifugation with Histopaque-1077 (density 1.077 g/mL, Sigma-Aldrich, St. Louis, MO, USA). MNCs were placed on fibronectin-coated 6-well plates in endothelial growth medium (EGM-2 MV, Cambrex, Walkersville, MD, USA) and cultured at 37 °C in a 5% CO_2_ incubator. After 2 to 4 weeks of MNC culture, a certain number of vascular progenitor cells continued to grow into colonies of late EPCs as late EPC-derived outgrowth endothelial cells.

### Tube formation assay

An EPC tube formation assay was performed using an in vitro angiogenesis assay kit (Chemicon, Temecula, CA, USA). EPCs transfected with miR-548j-5p mimic or antagomir were placed onto a matrix with medium for 16 h. Tubule formation was inspected by inverted light microscopy. Six random fields were used to calculate the average number of complete tubes formed by cells using Image-Pro Plus software (Media Cybernetics, Rockville, MD, USA).

### Scratch injury model and EPCs

Human EPC isolation, cultivation and characterization were performed as previously described^41^. EPC migration was evaluated using a scratch injury model. EPCs were transfected with miR-548j-5p mimic or antagomir, or a control. After serum-starvation of EPCs overnight, a scratch injury was applied with a scalpel, and EPC sprouting was examined before and 12/24 h after scratching.

### Directed in vivo angiogenesis assay (DIVAA)

Angiogenesis in vivo was evaluated using a DIVAA kit (Trevigen, Gaithersburg, MD, USA). Briefly, angioreactors were filled with EPCs transfected with 50,000 miR-548j-5p mimic, negative control, miR-548j-5p antagomir or scramble control and VEGF/FGF1 embedded in 20 μl of basement membrane extract^42^.

Angioreactors were incubated at 37 °C for 1 h. For positive controls, angioreactors were filled with BME supplemented with VEGF (12.5 ng/ml) plus FGF1 (37.5 ng/ml). Two angioreactors were implanted into each immunocompromised nude mouse (eight-week-old male) subcutaneously in the dorsal region. The angioreactors were removed 14 days after implantation and photographed. The presence of blood vessels was assessed using FITC-Lectin detection, and fluorescence was determined using a plate reader in mean relative fluorescence units.

### Western blot analysis

EPCs were lysed in a lysis buffer (62.5 mM Tris–HCl, 2% sodium dodecyl sulfate, 10% glycerol, 0.5 mM phenylmethanesulfonyl fluoride, 2 μg/mL aprotinin, pepstatin, and leupeptin), and proteins in the cell lysates were separated using sodium dodecyl sulfate–polyacrylamide (10%) gel electrophoresis, followed by transfer onto poly(vinylidene fluoride) (PVDF) membranes. The membranes were then probed with monoclonal antibodies against phosphorylated eNOS (Upstate Biotechnology, Lake Placid, NY, USA), SDF-1 and actin. Protein band densitometry was performed using ImageQuant software (Promega, Madison, WI, USA). According to the molecular weight of the target protein, the PVDF membrane was cut into several parts to allow three proteins to be analyzed simultaneously in the same gel.

### Ischemic hindlimb model and EPC transplantation

All animals were housed and handled in accordance with the criteria outlined in the National Institutes of Health Guide for Care and Use of Laboratory Animals. The study protocol was approved by the Institutional Animal Care and Use Committee of Taipei Veterans General Hospital, Taipei, Taiwan (approval number: IACUC_2013–076).

Eight-week-old male nude mice were purchased from BioLASCO Taiwan Co., Ltd. After 2 weeks of observation, the nude mice were then randomly assigned to 4 groups (n = 6 in each group) for intramuscular injection of EPCs transfected with miR-548j-5p mimic, miR-548j-5p mimic negative control, miR-548j-5p antagomir, or miR-548j-5p antagomir scramble control. Intramuscular injection was performed 24 h following the unilateral hindlimb ischemia surgery by left femoral artery ligation. A total volume of 5 × 10^6^ EPCs per animal in each group was injected at six sites into the ischemic hind limb distal to the arterial occlusion site. Three ventral injections were placed in the upper limb in proximity to the adductor and semimembranosus muscles, and the remaining three injections were administered to the ventral lower limb involving the gastrocnemius and flexor digitorum muscles. The blood flow of the hind limb was measured using a Laser Doppler perfusion imaging system (Moor Instruments Limited, Devon, UK) before and after surgery, and weekly thereafter. The results are expressed as the ratio of perfusion in the ischemic vs. the non-ischemic limb.

The mice were sacrificed 3 weeks after surgery and the limbs fixed overnight in methanol. The ischemic muscles were embedded in paraffin, and then deparaffinized, followed by incubation with rat monoclonal antibodies against murine CD31 (BD PharMingen, San Diego, CA, USA). New capillaries were identified based on morphology and positive staining for CD31 using the avidin–biotin complex technique and Vector Red Chromogenic substrate (Vector Laboratories, Burlingame, CA, USA) after counterstaining with hematoxylin. The visible capillaries were counted under 10 random fields, and the capillary density was expressed as the number of capillaries/mm.

### Statistical analysis

Data are expressed as the mean ± standard error of the mean (SEM). Statistical analysis was performed using the unpaired Student’s *t*-test or one-way analysis of variance (ANOVA), followed by Scheffe’s multiple comparison post hoc test using Statistical Package for Social Sciences software (version 14; SPSS, Inc., Chicago, IL, USA). *P* < 0.05 was considered to indicate a statistically-significant difference.

### Ethics approval and consent to participate

This study was approved by the Institutional Review Board for Research of Taipei Veterans General Hospital (approval number: 2013–08-020B#3), and the animal study protocol was approved by the Institutional Animal Care and Use Committee of Taipei Veterans General Hospital, Taipei, Taiwan (approval number: IACUC_2013–076). Written informed consent was provided by all participants prior to their inclusion in the study.

### Consent for publication

Not applicable.

## Supplementary Information

Supplementary Information.

## Data Availability

All data generated or analyzed during this study are included in this published article.

## Abbreviations

ABI: Ankle brachial index
CLI: Critical limb ischemia
eNOS: Endothelial nitric oxide synthase
EPC: Endothelial progenitor cell
FGF1: Fibroblast growth factor-1
miRNA: MicroRNA
NO: Nitric oxide
PAD: Peripheral artery disease
SDF-1: Stromal cell-derived factor-1
SEM: Standard error of the mean
VEGF: Vascular endothelial growth factor
