# Supplementary material for: miR-548j-5p regulates angiogenesis in peripheral artery disease
Source: Sci Rep. 2022 Jan 17;12:838. doi: 10.1038/s41598-022-04770-6 (PMC8764034; doi:10.1038/s41598-022-04770-6)
Supplement: Supplementary file 1 — Supplementary Information. [file 41598_2022_4770_MOESM1_ESM.pdf]

Supplementary Materials

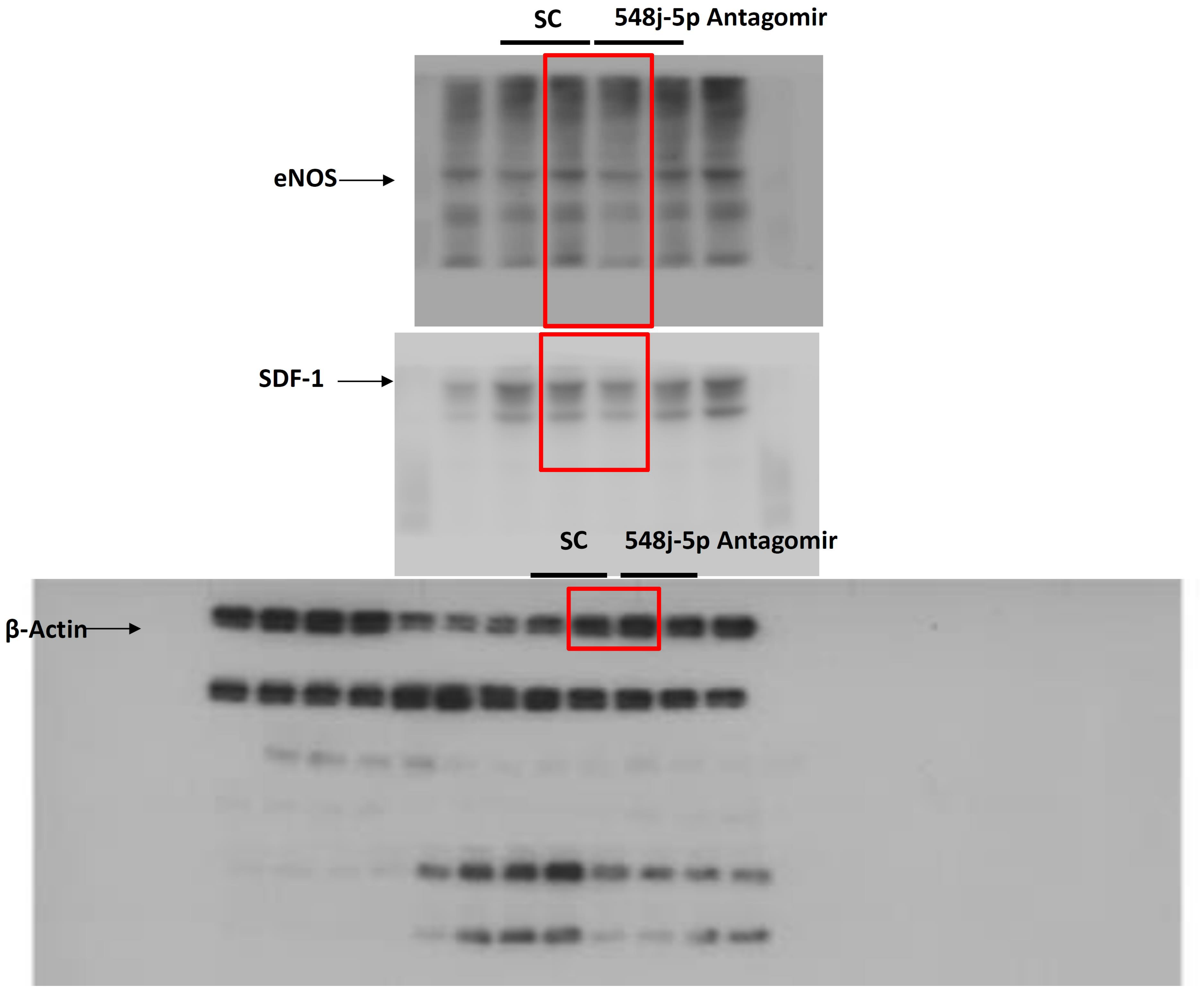

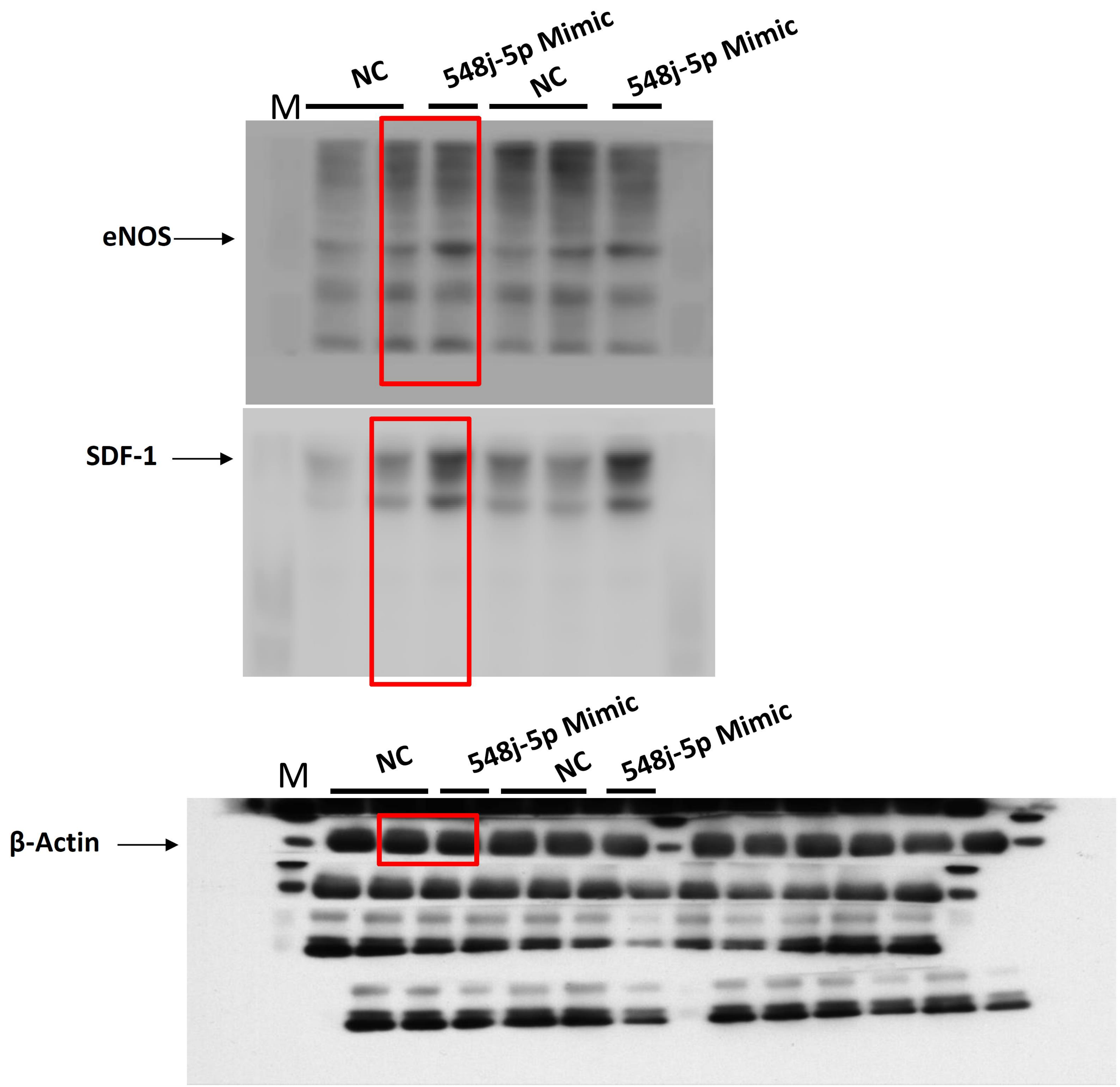

Supplement Figure 1: The full blot of the eNOs and SDF-1 protein bands that are included in Figure 5 of the main manuscript. SC: scramble control; NC: negative control; M:marker.
